# Digital screening for mental health in pregnancy and postpartum: A systematic review

**DOI:** 10.1007/s00737-024-01427-3

**Published:** 2024-04-01

**Authors:** Jocelyn R. Clarke, Melanie Gibson, Melissa Savaglio, Rhea Navani, Mariam Mousa, Jacqueline A. Boyle

**Affiliations:** 1 Monash Centre for Health Research and Implementation (MCHRI), Faculty of Medicine, Nursing & Health Sciences, Monash University, Melbourne, Australia; 2 Te Tātai Hauora o Hine – National Centre for Women’s Health Research Aotearoa, Wellington Faculty of Health,, Victoria University of Wellington,, Wellington, New Zealand; 3 Health and Social Care Unit (HSCU), School of Public Health and Preventive Medicine (SPHPM), Monash University, Melbourne, Australia; 4 Melbourne Health, Melbourne, Australia; 5 Health Systems and Equity, Eastern Health Clinical School,, Monash University, Melbourne, Australia

**Keywords:** Digital screening, Mental health, Pregnancy, Postpartum, Depression, Anxiety

## Abstract

**Purpose:**

This systematic review aimed to determine if digital screening for mental health in pregnancy and postpartum is acceptable, feasible and more effective than standard care (paper-and pen-based screening or no screening). The second aim was to identify barriers and enablers to implementing digital screening in pregnancy and postpartum.

**Method:**

OVID MEDLINE, PsycINFO, SCOPUS, CINAHL, Embase, Web of Science, Joanna Briggs Database and All EMB reviews incorporating Cochrane Database of Systematic Reviews (OVID) were systematically searched for articles that evaluated digital screening for mental health in pregnancy and postpartum between 2000 and 2021. Qualitative articles were deductively mapped to the Theoretical Domains Framework (TDF).

**Results:**

A total of 34 articles were included in the analysis, including qualitative, quantitative and mixed-methods studies. Digital screening was deemed acceptable, feasible and effective. TDF domains for common barriers included environmental context and resources, skills, social/professional role and identity and beliefs about consequences. TDF domains for common enablers included knowledge, social influences, emotion and behavioural regulation.

**Conclusion:**

When planning to implement digital screening, consideration should be made to have adequate training, education and manageable workload for healthcare professionals (HCP’s). Organisational resources and support are important, as well as the choice of the appropriate digital screening assessment and application setting for women. Theory-informed recommendations are provided for both healthcare professionals and women to inform future clinical practice.

## Introduction

Up to 20% of pregnant women are affected by mental health disorders such as depression and anxiety, during pregnancy or in the first year after giving birth (Bauer et al. 2014; PwC Consulting 2019). This has important implications for the mother’s mental health, infant attachment and wider family relationships, the mother’s partner or other children within the family unit (PwC Consulting 2019). Additionally, there are impacts on productivity through direct and indirect means (including healthcare costs), with total costs estimating up to $877 million dollars during the first year after birth in Australia (PwC Consulting 2019). Identifying and addressing mental health concerns in a timely manner with prompt and appropriate referral services for pregnant and postpartum women is vital.

There are several recommended validated measures for screening for perinatal psychological wellbeing in order to facilitate prompt referral and management for women at increased risk. Common validated screening tools include the Edinburgh Postnatal Depression Scale (EPDS) (Cox et al. 1987), Antenatal Risk Questionnaire (ANRQ) (Austin et al. 2013), Patient Health Questionnaire (PHQ-9) (Kroenke et al. 2001); Whooley Questions (Whooley et al. 1997) or General Anxiety Disorder Assessment (GAD-7) (Spitzer et al. 2006). Implementation of screening tools varies depending on the measures used and how they are implemented.

Perinatal mental health screening has primarily been undertaken as a clinical assessment or using paper-and pen-based assessments for validated measures, often conducted in clinics or during home visits. Barriers to perinatal mental health screening include limited mental health education and training for midwives and obstetricians, shortage of resources, time constraints, patient/provider interaction, and systems level issues such as cost and location (Kim et al. 2010; Byatt et al. 2012). In addition, pen and paper-based assessments are often time consuming and prone to scorer error between 13.4% and 28.9% (Matthey et al. 2012), and may involve time delays for processing reports within a clinic setting.

Digital screening for mental health in pregnancy and postpartum may provide a way to save time, reduce scorer error and increase referral and treatment for mental health issues. Digital health is increasingly being incorporated in health services across the world, can facilitate sharing of health information between patients and health professionals and across health systems and can support decision making with built in algorithms and local care pathways (Bernabe-Ortiz et al. 2008; Paperny et al. 1990; Quispel et al. 2012). Digital screening as defined in this systematic review is the use of valid and reliable screening tools such as the EPDS (Cox et al. 1987) or ANRQ (Austin et al. 2013) used in digital or electronic format (e.g., mobile phone, tablet, laptop, desktop computer, through mobile applications or web link) completed by women in pregnancy and postpartum (up to 36 months).

To the authors’ knowledge, there are no systematic reviews that explore digital screening for mental health in pregnancy and postpartum. Therefore, this review aims to determine if digital screening for mental health in pregnancy and postpartum is acceptable, feasible, and more effective than standard care (e.g., paper-based psychological assessments; no screening). Effective screening accurately detects symptoms of mental health conditions in pregnancy and postpartum (or accurately identifies women at elevated likelihood of currently experiencing a mental health condition), leading to an appropriate referral for further assessment being made. In practice, this usually means screening for depression and anxiety as recommended in clinical guidelines as the most common mental health conditions in the perinatal period. Feasibility results in quicker administration time, increased screening capacity, reduced scoring error, generated individual tailored clinical and patient reports, prompted referrals for the treatment of depression and anxiety and technology accessible and easy to use. Acceptability and feasibility will be determined by information reported by both women and healthcare professionals (HCPs) and effectiveness will be determined by good internal consistency (e.g., Cronbach’s α), comparison groups and cross-cultural considerations. For this review, HCPs refers to professions involved in women’s perinatal care, including doctors, midwives, obstetricians, nurses, psychologists, and psychiatrists.

This systematic review also aims to determine what the barriers (e.g., challenges) and enablers (e.g., facilitators) are to implement digital screening for mental health in pregnancy and postpartum and provide recommendations for best practice digital perinatal mental health screening.

## Method

### Search strategy and selection criteria

A study protocol was registered with PROSPERO (CRD42020198372) (https://www.crd.york.ac.uk/prospero/#recordDetails). The search located research literature within the last 21 years (from 2000 to 2021) on digital screening for mental health in pregnancy and postpartum. This time period was chosen to reflect the introduction of digital technology around the world. This review placed no restrictions on language. In total, eight databases were searched: OVID MEDLINE, PsycINFO, SCOPUS, CINAHL, Embase, Web of Science, Joanna Briggs Database and All EMB reviews incorporating Cochrane Database of Systematic Reviews (OVID). The search strategy included terms for digital health, screening, mental health, pregnancy and postpartum, including but not limited to mobile technologies, self-report, psychiatric disorder, peripartum period and postpartum period. A full list of search terms is available in Supplementary File 1). The end date of the search was 23rd July 2021.

Studies were included if they met the following criteria: (1) participants were (a) women of birthing age or had given birth or were currently pregnant or (b) Healthcare Professionals; (2) mental health screening was conducted using digital technology (e.g., tablets, mobile phones, online survey link, computers) and validated tools (e.g., EPDS); (3) have comparison groups including no screening, paper-based screening, clinical assessment only, non-validated symptom assessment, psychosocial assessment without symptom assessment, or no comparison group; (4) outcomes included barriers and enablers to digital screening within the Theoretical Domains Framework (Cane et al. 2012); acceptability, feasibility, effectiveness, efficiency, cost and sustainability of digital screening; symptoms of anxiety or depression; presence of psychosocial risk factors, with the proportion of women meeting threshold scores to be considered at risk; mean or median scores; (5) study type included systematic reviews (with or without meta-analysis) with a quality or risk of bias assessment; longitudinal cohort studies; cross-sectional studies; case control studies; qualitative studies; evaluations; medical records audits; administrative data; randomised control trials and before and after studies and (6) included all languages, information after the year 2000 and no sample size limit.

The use of paper-based psychological assessments or clinician administered assessments uploaded into the Electronic Medical Record (EMR) or Electronic Health Record (EHR) were not considered as digital screening for this systematic review. Clinical decision support systems, algorithms and machine learning were only included if they involved the use of a psychological assessment in digital format.

Studies were excluded if they met the following criteria:

1. The study explored only men’s or father’s experiences
2. The study only included women with post-traumatic stress disorder or other pre-existing mental health issues
3. Used non-validated tools
4. Had family violence as a sole outcome measure
5. Were a conference abstract, commentary, editorial, narrative review, position statement or a non-research letter

### Metric definitions

#### Acceptable intervention

 – determined as reported by women and HCP’s.

#### Feasible intervention

– determined by quicker administration time, increased screening capacity, reduced scorer error, generated individual tailored clinical and patient reports and prompted referrals for the treatment of depression and anxiety, technology accessible and easy to use.

#### Effective intervention

 – determined by accurately detecting symptoms of depression and anxiety in pregnancy and postpartum (or accurately identifies women at an elevated likelihood of currently experiencing depression and anxiety), leading to an appropriate referral being made. Will be determined by good internal consistency (Cronbach’s α), comparison groups and cross-cultural considerations.

### Study selection

One author (JC) independently assessed the title, abstract, keywords and full-text of every article retrieved against the defined selection criteria. Two authors (RN & MS) shared the role of second reviewer of all studies that met criteria. Any disagreement at both the title and abstract review and full-text review stage was resolved by discussion with the second reviewers to achieve 100% consensus.

### Data extraction

The study characteristics for the 34 full-text articles included author and year, country and setting, ethnicity, study population, sample size, age of participants, research objectives, recruitment strategy, key inclusion and exclusion criteria of the studies, digital mode, methodological and theoretical approach, method and duration of data collection, data analysis, study design, rate of attrition, study findings, outcomes and power calculations, preliminary TDF domain and quotes from qualitative and mixed-methods studies. Data extraction information was recorded on one Excel spreadsheet. Five authors from the included studies were contacted for additional information and added to the data extraction.

### Risk of bias assessment

One author (JC) independently assessed risk of bias using assessment templates suitable for the included studies, including the Critical Appraisal Skills Programme template for qualitative studies (CASP 2018), MCHRI risk of bias templates for quantitative studies (MCHRI 2013; MCHRI 2014) and the Mixed Methods Appraisal Tool (MMAT) risk of bias template for the mixed methods and remainder of the studies (e.g., Quantitative – Descriptive pre-post-test) (Hong et al. 2018). The risk of bias templates assessed the studies’ internal and external validity such as the use of appropriate study design, inclusion and exclusion criteria, reporting bias, confounding, sufficient power analyses and any conflicts of interest. Using a descriptive approach, the studies were given a rating of low, moderate, high or unclear risk of bias. Twenty percent of the studies (7 articles) were reviewed by author MM. Authors JC and MM discussed the risk of bias and evaluation methods used until 100% consensus was reached.

### TDF framework

The Theoretical Domains Framework (TDF) (Cane et al. 2012) provides an integrative theoretical framework for the evaluation of behaviour change and implementation across disciplines within the healthcare industry. It comprises of 14 key domains (e.g., Knowledge) with 83 constructs. The findings of the systematic review were mapped to the TDF domains and constructs to identify the barriers and enablers digital screening has for mental health in pregnancy and postpartum for both women and HCP’s.

The TDF framework is a valid framework that explores both individual and organisational aspects of implementation research and is effective in providing theory informed and evidence-based support for healthcare interventions (e.g., Michie et al. 2008; Cane et al. 2012; Francis et al. 2012; French et al. 2012).

It has been used previously to evaluate perinatal mental health screening (Nithianandan et al. 2016) (Table 1 and 2).

**Table 1:** Summary of the study characteristics of the 34 included full-text articles in systematic review

ID	Author/ Year	Country	Study Population	Sample Size	Setting	Research Objectives & Research Question	Digital Mode & Method & other assessments	Methodological/ Theoretical approach	Data Collection	Data Analysis	Study Duration	Risk of bias
1	Bante et al. (2021)	Arba Minch Zuria district, Gamo zone; Southern Ethiopia	Pregnant women	667	Community; Public Health; Public University	To assess Comorbid Anxiety and Depression (CAD) and associated factors among pregnant women Helping to understand the prevalence of comorbid anxiety and depression	PHQ-9; GAD-7 collected using Open Data Kit (ODK) android application; Women’s Abuse Screening Test (WAST); Household Food Insecurity Access Scale (HFIAS)	Theoretical and practical training approach for data collectors; Community-based Cross-Sectional Study Design	Data collectors were used to collect data from participants via the Open Data Kit (ODK)	Socio-demographic (frequency & percentage) and socioeconomic characteristics; obstetric characteristics (frequency & percentage); prevalence of comorbid anxiety and depression (percentage); factors associated with comorbid anxiety and depression (N, %, OR, p-value, Adjusted Odds Ratios; 95% CI, p-value)	11 months	Moderate
2	Barry et al. (2017)	Ireland & England, UK	Pregnant women	21	Public Health	Virtue ethics for mHealth design; Self-report during pregnancy Helping to understand the barriers and enablers of mHealth design	EPDS-10; EMA; BrightSelf App; self-report	Qualitative	Case Study; Individual design sessions; Group design sessions	Thematic Analysis	5 Design Sessions	Low/Moderate
3	Diez-Canseco et al. (2018)	Lima, Peru	Women (Antenatal care service)	931	Public Health; Primary Health Care	Design, develop and test an intervention to promote early detection, referral and access to treatment of patients with mental health issues in public primary health care Helping to understand the feasibility and effectiveness of digital screening	SRQ (WHO) – 28 questions (Peru version); mHealth screening App	Mixed-Methods	Qualitative and Quantitative data collected concurrently	Quantitative: descriptive analyses, frequencies and percentages; Qualitative: Interviews	9 weeks (Healthcare Provider Training)	Moderate
4	Doherty et al. (2020)	London & Cambridge, UK	Women (pregnant & non-pregnant) and Health Professionals	38	Public Health	Clinical interface of a mobile application for the self-report of psychological wellbeing and depression during pregnancy Helping to understand the barriers and enablers of digital screening	EPDS-10; BrightSelf App; self-report	Qualitative; Tatar’s Design Tensions Framework	Design sessions with women and Health Professionals (one of five large group design sessions or one of 17 individual sessions)	Thematic Analysis	5 Large group design sessions; 1 of 17 individual design sessions	Low/Moderate
5	Doherty et al. (2018)	London & Cambridge, UK	Women (pregnant n = 8 & non-pregnant n = 3) and Health Professionals (n = 27)	38	Public Health	To explore the issues and challenges surrounding the use of mobile phones for the self-report of psychological well-being during pregnancy Helping to understand barriers and enablers of digital screening	EPDS-10; BrightSelf App; self-report	Qualitative; Tatar’s Design Tensions Framework	Individual design sessions; Group design sessions; Skype design sessions	Thematic Analysis	Individual design sessions; 5 Group design sessions; 6 Skype design sessions	Low/Moderate
6	Drake et al. (2014)	United States (Southern)	Women (Postnatal); healthy volunteers	18	Health Sciences Centre; Public Health	To develop innovative methods of screening women for the symptoms of PPD to facilitate referral and treatment Helping to understand the barriers and enablers of digital screening; helping to understand the efficacy, feasibility and acceptability of digital screening	EPDS-10 (online/Internet); Laptop	Mixed-Methods (Descriptive); Exploratory; Qualitative methods	Focus Groups; Individual interviews; Online screening intervention	Thematic Analysis	Self-administered EPDS 2–3 months postpartum	Low/Moderate
7	Dyurich & Oliver (2020)	South Texas; United States	Women (pregnant)	6	Maternal–fetal Clinic	To explore the lived experiences of pregnant women using an electronic intervention to screen for and manage symptoms of perinatal depression and promote wellness during pregnancy Helping to understand the barriers and enablers of digital screening	EPDS-10; VeedaMom mobile App	Qualitative – Individual; Phenomenological Study	Lived experience; in App journal; Semi-structured interviews; Focus Groups; preliminary themes	Thematic Analysis; Focus Groups	EPDS completed once a week for 6 weeks	Low/Moderate
8	Faherty et al. (2017)	Philadelphia, Pennsylvania, United States	Women (prenatal)	36	University Hospital	To examine, using a smartphone application, whether mood is related to daily movement patterns in pregnant women at risk for perinatal depression Helping to understand the feasibility of digital screening to monitor perinatal depression	Application administered surveys (Ginger.io) (PHQ-2 (daily) & PHQ-9 or GAD-7 administered weekly	Quantitative; Cohort Study (ecologic momentary assessment; randomised; Cohort)	Enrolment interview; Data collection via Ginger.io Application (PHQ-2; PHQ-9; GAD-7); mobility and radius data	Demographic factors compared between mild/moderate and moderately severe/severe depression at baseline; General linear mixed-effects regression models to estimate the association between mood and movement	8-weeks	Moderate
9	Flynn et al. (2011)	Ann Arbor, Michigan, United States	Pregnant (n = 81) and Postpartum Women (n = 104)	185	Outpatient Psychiatry Clinic; University affiliated health care system	To compare the utility of the EPDS with the PHQ in a sample of perinatal women seeking psychiatry services within a large health care system Helping to understand the effectiveness of digital screening	Computerised versions of the EPDS-10 & PHQ-9 (PHQ-9 used a summary scoring algorithm and a diagnostic algorithm)	Quantitative – Non-RCT	Extracted archival data; EMR; unstructured Clinical Interview using DSM-IV by Clinician	Quantitative Analysis: Pearson correlations; Cronbach's coefficient alphas; Comparative AUC for ROC contrasts between EPDS and PHQ	2 years and 3 months (extracted archival data)	Low
10	Fontein-Kuipers & Jomeen (2019)	Rotterdam, The Netherlands	Dutch-speaking pregnant women with uncomplicated pregnancies	433 (T1) 343 (T2)	Primary Care (Midwife- led)	To investigate the validity and accuracy of the Whooley questions for routine screening of maternal distress in Dutch antenatal care Helping to understand the effectiveness of digital screening	Whooley Questions (2-items); Arroll Question 1 question); EDS-10; STAI (20-items); PRAQ-R2 (10-items) (self-completed and digitally distributed) (Dutch version)	Quantitative – Cohort Study	Data collected digitally via self-report measures	Quantitative Analysis; proportion of maternal distress; reliability analysis of Whooley questions; diagnostic accuracy of Whooley items for depression, trait-anxiety, pregnancy-related anxiety; population prevalence of maternal distress; ROC analysis of EDS, STAI and PRAQ-R2 at T1 & T1 (Q1 &2)	1 year and 11 months (data collection)	Moderate
11	Friedman et al. (2016)	East Harlem, New York, United States	Health Professionals (Pediatric Residents & Faculty); Mothers	Health Professionals (Pre-test n = 40; Post-test n = 30; Post-test who attended Conference (n = 17); Mothers in Chart Review (Group 1: 100; Group 2: 100; Group 3: 93)	Medical Centre	The study examined the effects of an educational session about PPD and modification of the electronic medical record (EMR) on providers’ screening for PPD Helping to understand the effectiveness of digital screening	EMR; PHQ-2 (Researchers integrated a screening tool into the EMR to screen for PPD; EMR template change)	Quantitative – Descriptive (pre-test-post-test Study Design)	Retrospective Chart Review of Mothers	Data were analysed using chi-square tests and Student’s t tests; Pre- & Post-test sample sizes and percentages	Retrospective Chart Review; Three time periods: Group 1 = before the conference; Group 2 = after the conference but before the EMR change and Group 3 = after screening in the EMR	Low
12	Gance-Cleveland et al. (2019)	Aurora, Colorado, United States	Prenatal providers; Prenatal patients; Clinicians – nurse-midwives; obstetrician, family nurse practitioner; certified nurse-midwife administrators	Prenatal providers (n = 9); Prenatal patients (n = 7); Clinicians – nurse-midwives (n = 7); obstetrician (n = 1), family nurse practitioner (n = 1); certified nurse-midwife administrators (n = 2)	Midwifery Clinic	To develop StartSmart, a mobile health (mHealth) intervention to support evidence-based prenatal screening, brief intervention, and referral to treatment for risk and protective factors in pregnancy Helping to understand the enablers and barriers of digital screening	GAD-2; GAD-7; PHQ-2; PHQ-9; AAS-2; NIDA Quick Screen; AUDIT-C; Pre-pregnancy BMI or GWG; GTT; Godin-Sheperd; Insomnia Severity Index	Qualitative – mHealth Development approach; Davis’ Technology Acceptance Model; Screening, Brief Intervention, Referral to treatment (SBIRT) framework; Cognitive engineering method	Interviews; Qualitative observations; Process Mapping; Focus Groups; Online Advisory Work Groups	Phase 1: Prototype development; Phase 2: Alpha testing; Clinician and patient testing and feedback	First prenatal visit; 28-week visit and 36- week visit	Low/Moderate
13	Gordon et al. (2016)	Philadelphia, Pennsylvania, United States	Patients with history of depression in pregnancy; Prenatal providers; Social Workers/Care Managers; Mental health Specialists; Clinic Administrator; Support staff; Research staff; and a Programmer	Patients with history of depression in pregnancy (n = 4), Prenatal providers (n = 2), Social Workers/Care Managers (n = 2), Mental Health Specialists (n = 2), Clinic Administrator (n = 1), Support staff (n = 3), Research staff (n = 2), and a Programmer (n = 1)	Large hospital-based Outpatient Prenatal Care Centre	To develop a suite of eHealth applications to improve the quality of perinatal mental health care Helping to understand the feasibility of digital screening to screen for perinatal depression	Tablet-based, self-report screening tool PMD) using a 2-stage process with an initial 2-question screen & PHQ-9	Qualitative – Participatory Design (Longitudinal); a rapid cycle iterative design approach	Participatory Groups; Feedback; Live action videography; Field Notes	Longitudinal Participatory Design approach; Development of 3 applications	20 meetings over 24 months	Low/Moderate
14	Guevara et al. (2016)	Philadelphia, Pennsylvania, United States	Clinicians; Parents	Clinicians (n = 15); Parents (n = 1,816)	Hospital affiliated paediatric practices and Community Health Clinics	To determine feasibility and acceptability of parental depression screening in high-risk urban paediatric practices Helping to understand the feasibility and acceptability of digital screening; helping to understand the barriers and enablers of digital screening	EHR; electronic alerts/point of care reminders for Clinicians; electronic versions of the PHQ-2; automated scoring algorithm; suggested language for explaining positive screen to parents	Mixed-Methods (Qualitative & Quantitative components); Grounded Theory	Rates of depression screening using PHQ-2; Semi-structured interviews with Clinicians to identify barriers and facilitators to screening; Investigator Meetings; Screening of parents was conducted when they brought their child to the practice or clinic for a well child visit between the ages of 12 months and 36 months	Summary statistics on the number of eligible parents, depression screens administered, and positive screens by site were collected; Differences in proportions by site using chi- square statistics; Assessed for trends in the monthly proportion screened using a chi- square test of trend statistic; Thematic Analysis	20-month screening period	Moderate
15	Guintivano et al. (2018)	North Carolina, United States; Australia	Women	7344 (women with lifetime history of PPD) (US); 411 (Australia)	Lifetime episode of having PPD (US & Australia) (General Population)	To develop an iOS App (PPD ACT) to recruit, consent, screen, and enable DNA collection from women with a lifetime history of PPD to sufficiently power genome-wide association studies Helping to understand the effectiveness of digital screening for PPD	EPDS-lifetime (modified version; 21 questions) to assess lifetime history of PPD; 2nd EPDS assessment used a web-based form; PPD ACT	Quantitative – Cohort Study	Online screening for PPD depression symptoms using EPDS; Clinician diagnosis; Spit Kits; Biobanking	Descriptive statistics; State-level birth rate data; ICC’s to measure test–retest reliability for continuous variables; Binomial tests to measure agreement for binary variables; Squared weighted Cohen’s kappas to measure test–retest reliability for categorical variables	1 year	Moderate
16	Hahn et al. (2021)	Aachen, Germany	Women (Mothers; Postpartum)	Cohort 1 (N = 308); Cohort 2 (N = 193)	University Hospital	To explore whether an accurate prediction of PPD is feasible based on socio-demographic and clinical-anamnestic information as well as early symptom dynamics using remote mood and stress assessments Helping to understand the feasibility and acceptability of digital screening	EPDS collected via remote online questionnaires sent via email; collected at all time points (T0-T4); personal and socio-demographic variables; Stressful Life Events Screening Questionnaire; Maternal Postnatal Attachment Scale (MPAS)	Quantitative – Cohort Study (Cohort 1 & 2); Longitudinal	Data collected at Clinic and remote online assessments (T0-T4); mood and stress assessments collected on a bi-daily basis; clinic assessments; clinical interview	Univariate analysis (χ^2^, N and p-value) of the first cohort; Logistic regression coefficients; Socio-demographic variables; birth complications; subjective birth-related trauma; PMS; postpartum blues; stressful life events; breastfeeding; within- and out-of-sample validation study design	12 weeks (data collection)	Moderate
17	Hassdenteufel et al. (2020)	Heidelberg, Germany	Women (pregnant)	597	University Hospitals – Maternity Departments	To examine the longitudinal interaction between exercise, general physical activity, and mental health outcomes in pregnant women Helping to understand the feasibility of digital screening	EPDS-10; PRAQ-R (10-items); STAI-S; STAI-T (20 questions each) & physical activity levels using PPAQ (32 activities); Global Health Scale (GHS) (10 questions); completed on Tablets or Computers via self-report	Quantitative – Cohort Study (Prospective Longitudinal Study)	Online screening; Self-report	Cross-sectional and longitudinal analyses using Pearson’s correlation coefficient and multiple linear regression analyses	Digital assessment every 4 weeks from 2nd trimester until birth, as well as 3 & 6 months postnatally (1-year, 23 months data collection)	Low
18	Highet et al. (2019)	Melbourne, Australia	Women (pregnant)	144	Maternal and Child Health Clinic	To evaluate a perinatal mental health digital screening platform, iCOPE Helping to understand the effectiveness of digital screening	EPDS-10; psychosocial risk questions; iCOPE Digital Screening	Quantitative – Cohort Study (Descriptive)	iCOPE Digital Screening platform automatically recorded and scored the EPDS; produced instant clinical and client reports whilst collecting data in real time	Participant characteristics; psychosocial risk (n & %); mean screening time; rates of depression and anxiety (Cronbach’s α for EPDS administered digitally)	12-month period (4–6-week postnatal check)	Moderate
19	Jiménez-Serrano et al. (2015)	Valencia, Spain	Women (postpartum)	No PPD: n = 1,237; PPD: n = 160	General Hospitals	To develop classification models for detecting the risk of PPD during the first week after childbirth, enabling early intervention and to develop an mHealth App for mothers and clinicians to monitor their results Helping to understand the effectiveness of digital screening	EPQ-N (12-items); EPDS-10; Machine Learning; Risk Prediction; Mobile Phone App; eDPP Predictor	Quantitative – Cohort Study (Prospective)	Digital screening; Diagnostic Interview	Machine Learning (ML); Pattern Recognition (PR); Naive Bayes Model; Logistic Regression; artificial neural network (ANN); support vector machines (SVM)	11-month period (at childbirth; Week 8 and at Week 32 after childbirth)	Moderate
20	Johnsen et al. (2018)	Copenhagen, Denmark	Women (pregnant)	15	Antenatal Care Facility (1st Midwifery visit)	To explore women's experiences of self-reporting their health status and personal needs online prior to the first midwifery visit, and how this information may affect the meeting between the woman and the midwife Helping to understand the barriers and enablers of digital screening	Email link to a self-report Questionnaire; socio-demographic characteristics, reproductive, obstetric, and medical history, general health status, intake of dietary supplements, lifestyle factors before and during current pregnancy, WHO-5 Well-being Index, and Cambridge Worry Scale	Qualitative	Individual semi-structured Interviews; Structured observations of first midwifery visit	Conventional Content Analysis was used to analyse data; categories developed (main and sub-categories)	15th gestational week (1st midwifery visit); 1 year of data collection	Low
21	Kallem et al. (2019)	Philadelphia, Pennsylvania, United States	Women	195 (Received Services (n = 23) Did Not Receive Services (n = 172)	Urban Primary Care Practice (2-month Well Child Visit)	To determine mental health care use among women with Medicaid insurance 6-months after a positive PPD screen and to determine maternal and infant factors that predict the likelihood of mental health care use Helping to understand the effectiveness of digital screening	EPDS-10 (English & Spanish); Tablet Screening in waiting room; Self-Report	Quantitative – Retrospective, Population-based Cohort Study	A linked dataset of the child’s electronic health records, which includes the PPD screens of Mothers, maternal Medicaid claims, and birth certificates were used	Bivariate analyses (Chi-square and t test) were conducted comparing the maternal and infant factors of mothers who completed the EPDS and did not complete the EPDS; Multivariate logistic regression was used to estimate maternal and infant clinical and sociodemographic factors that predict service use	2-month Well Child Visit; 2 years and 11 months (data collection)	Moderate
22	Kim et al. (2007)	Minneapolis, Minnesota, United States	Women (prenatal)	54	Medical Centre (University affiliated Public Hospital) (routine prenatal visit)	To test the feasibility of using Interactive Voice Response (IVR) technology to screen for depression among low-income, urban pregnant patients and to solicit their preferences for treatment Helping to understand the acceptability and feasibility of digital screening	Interactive Voice Response (IVR) technology; automated phone version of the EPDS-10; Treatment Module (7 questions)	Quantitative – Cohort Study; convenience sample; pilot study	IVR—Introduction module; Depression screen module & Treatment module	Quantitative outcomes of interest were completion rates for the IVR screening and the percentage of women with mild to severe depressive symptoms. Research outcomes included reports of patient satisfaction (n & %) with the system along with their preferences for an intervention	One-month study period; two different weekly prenatal clinics	Moderate
23	Kingston et al. (2017)	Edmonton, Alberta, Canada	Women (pregnant)	N = 636; Paper-based screening group n = 331; E-Screening group n = 305	Community and Hospital-based Antenatal Clinics and Hospital-based prenatal classes (Maternity Clinics)	To evaluate the feasibility and acceptability of Web-based mental health e-screening compared with paper-based screening among pregnant women and to identify factors associated with women’s preferences for e-screening and disclosure of mental health concerns Helping to understand effectiveness over paper; helping to understand the feasibility and acceptability of digital screening	Web-based mental health e-screening; The intervention group completed the ALPHA (15 risk factors) and the EPDS-10; Tablet computer; Women in the Control Group completed paper-based versions of the ALPHA and the EPDS, followed by the web-based baseline questionnaire; MINI	Quantitative—Parallel-group, Randomized Controlled Superiority Trial	E-Screening Intervention; Paper-Based Screening Control Group	Adapted version of Renker and Tonkin’s tool of feasibility and acceptability; ITT analysis; Baseline differences in groups were compared using independent t tests (means) and chi-square tests (%); Descriptive data (frequencies and 95% CIs; means and SDs) to describe the sample	1 year 5 months (data collection)	Low
24	Lupattelli et al. (2018)	Western Europe; Northern Europe; Eastern Europe	Women (Antenatal and Postnatal)	8069	Online (Anonymous)	To explore the prevalence of self-reported antenatal and postnatal depressive symptoms by severity across multiple countries and the association between antidepressant treatment in pregnancy and postnatal symptom severity Helping to understand the prevalence of antenatal and postnatal depression	EPDS-10; Electronic questionnaire; Questback	Quantitative – Cross-Sectional Study	Data were retrieved from the “Multinational Medication Use in Pregnancy Study,” a cross-sectional, web-based study carried out in Europe, North and South America, and Australia to investigate patterns and correlates of medication use in pregnancy	Descriptive statistics; IPTW, using the propensity score to survey data; logistic regression; crude and adjusted β coefficients with 95% CI	5 months (data collection)	Moderate
25	Marcano-Belisario et al. (2017)	England, United Kingdom	Women (pregnant)	530	General Practice, Community or Hospital centres (NHS) Antenatal Clinics	To assess the feasibility of using tablet computers in the waiting area of antenatal clinics for implementing the recommendations of the NICE guidelines for recognising antenatal depression Helping to understand the feasibility of digital screening	Whooley questions (2-items); EPDS-10; Socio-demographic survey (11 questions); Tablet computers; scrolling and paging format; Snap Mobile App	Quantitative – Randomised Controlled Trial	Use of tablet computers to collect socio-demographic data; complete Whooley & EPDS items; survey layout (scrolling and paging); Snap WebHost	Completion times (median, mins, secs); proportion; median; chi-square; sample sizes and percentages	8 months (recruitment of participants)	Moderate
26	Pineros-Leano et al. (2015)	Illinois, United States	Staff members (7 nutritionists; 5 nurses; 3 case managers, 3 administrative Assistants; 3 intake specialists; 4 program coordinators	25	Public Health Clinic	To explore the attitudes and perceptions staff members towards incorporating mHealth technology in a public health clinic to screen for depression Helping to understand the barriers and enablers of digital screening	Staff perceptions related to depression screening with tablet technology	Qualitative	Focus Groups; Semi-structured interview guide; audio recorded; transcribed verbatim	Thematic Analysis (Focus Group Data)	1 month (data collection)	Low
27	Poleshuck et al. (2015)	New York, United States	Women (pregnant/non-pregnant)	159	Women’s Health Clinic	To determine the feasibility and acceptability of an electronic psychosocial screening and referral tool; developed and finalized a prioritization tool for women with depression; and piloted the prioritization tool Helping to understand the acceptability and feasibility of digital screening	An electronic psychosocial screening and referral tool; Promote-W uses primarily standardized screening tools; PHQ-9; a tablet computer with the Patient Navigator in the clinic; WHO-QOL scale; Client Satisfaction Questionnaire (patient satisfaction)	Quantitative – Clinical Trial/randomized comparative effectiveness—RCT	Community Advisory Board; Focus Groups; Individual patient input	Analytic plan—growth curve analysis; quadratic effects; cross-sectional mean differences using ANCOVA; moderation effects; latent class analysis	Participants are assessed at baseline, at 4-months immediately post-treatment, and at 3- and 6-months following the end of treatment at any safe location of their preference, or by phone if necessary	Low
28	Quispel et al. (2012)	Rotterdam, The Netherlands	Women (pregnant)	621	Obstetric Clinic (University Hospital); Community Midwifery Practice	To explore the reliability, validity (predictive value) and feasibility of the GyPsy approach under routine practice conditions in Rotterdam, the Netherlands Helping to understand the effectiveness and feasibility of digital screening	EDS-10 (Dutch version); GyPsy Screen and Advice; Self-report questionnaire; PDA	Quantitative – Cohort Study (Observational & Exploratory)	PDA questionnaire; caregiver showed screen result and provided women advice or provided other specific care	Cronbach’s α coefficient; intraclass correlation coefficient, Cohen’s κ and Kendall’s τ-b. Criterion validity NPV; PPV secondary measure; risk profiles and to describe feasibility judgements they used conventional descriptive and comparative statistics; Posthoc Bonferroni adjusted pair wise comparisons were performed to identify any group related difference; Power 0.80 and p value < 0.05	1 year 11 months (data collection); 43 women completed retest of EDS	Moderate
29	Martinez-Borba et al. (2019) within Cipresso, Serino & Villani (2019)	Spain	Women (perinatal)	523	Health Collaborating Centres; Community recruitment	To compare the feasibility, usability, and user satisfaction of two devices (web vs. mobile application) of an online program for perinatal depression screening called HappyMom (HM) Helping to understand the acceptability and feasibility of digital screening	EPQ-R (48-items), STAI-T (20-items), ERQ (10-items), CAE (42-items), QLI (33-items) and SRSS (43-items); HM-Web and HM-App	Quantitative – Longitudinal; Cohort Study	Two evaluations were made during pregnancy (weeks 16–24 and 30–36 of gestation) and three in the postpartum (weeks 2, 4, and 12 after delivery). The assessment points were the same for both devices	Descriptive analysis of the sample; Analysis of dropout rates (proportion of women who completed each assessment in relation to women who were registered into the program); Exploration of women’s usability reports and satisfaction with HM	4 years (data collection)	Moderate
30	Shore et al. (2020)	Colorado, United States	Women (perinatal)	135 (referred patients)	Women’s Clinic	To describe the implementation of the first known telepsychiatry-enabled model of perinatal integrated care and to report initial results following implementation Helping to understand the effectiveness of digital screening	PHQ-9; EPDS; Tablet computer	Quantitative – Cohort Study; Quality Improvement Study; descriptive design; convenience sample; pilot study	PHQ and EPDS completed electronically on a tablet computer; demographic data and diagnoses; satisfaction surveys; biannual reports; EHR	Descriptive analyses on patient characteristics, process measures and outcome measures (%, N, χ^2^,df, p-value)	14 months (data collection); Satisfaction surveys were distributed to a convenience sample of patients in September 2017 and July 2018	Low/Moderate
31	Tsai et al. (2014)	Khayelitsha, Cape Town, South Africa	Women (pregnant)	Study 1 N = 1,144 and Study 2 N = 361; Total N = 1,505	Community Health	To determine the extent to which community health workers could also be trained to conduct case finding using short and ultrashort screening instruments programmed into mobile phones Helping to understand the effectiveness and feasibility of digital screening	EPDS-10 (Xhosa version); Mobile Phone; Survey software	Quantitative—Cross-Sectional Study (× 2)	EPDS completed on a mobile phone (EPDS-7, EPDS-5, EPDS-3, EPDS-2)	Cronbach’s α coefficient; Pearson correlation coefficient; calculating sensitivity, specificity, and likelihood ratios using standard formulas; ROC curves, calculating the area under the ROC curve (AUC) using the trapezoidal rule and comparing AUC values using the algorithm	Study 1—These data were collected from May 13, 2009 to September 29, 2010 in 24 non-contiguous neighbourhoods of Khayelitsha; Study 2—May 1, 2010 through February 18, 2011	Moderate
32	Willey et al. (2020)	Melbourne, Australia	Women (pregnant) refugee and migrant	N = 22; refugee background (n = 17) migrant (n = 5) backgrounds	Antenatal Clinic	To determine if a digital perinatal mental health screening program is feasible and acceptable for women of refugee background Helping to understand the feasibility and acceptability of digital screening	EPDS-10; iCOPE	Qualitative – Evaluation Study	Focus Groups; Semi-structured interviews; use of Interpreters to assist women who couldn't speak much English	Thematic analysis – inductive and deductive approach; saturation of themes; hybrid approach to thematic analysis was utilised	4 months (data collection)	Low
33	Woldetensay et al. (2018)	Ethiopia (South-Western—rural)	Women (pregnant)	4680	Community	To describe the prevalence of prenatal depressive symptoms and whether it is associated with maternal nutrition, intimate partner violence and social support among pregnant women in rural Ethiopia Helping to understand the prevalence of prenatal depressive symptoms	Depressed mood was assessed using PHQ-9; MUAC; HemoCue Hb 301 system; Household Food Insecurity Access Scale; Socio-demographic variables; Obstetric factors; IPV (HITS assessment); MSSS; Data collection was conducted electronically using ODK software; handheld tablets; submitted to a secured server via an internet connection	Quantitative – Cohort Study (Prospective, Community based, Birth Cohort Study—Open; Quasi-Experimental)	Data collection was conducted electronically on handheld tablets and submitted to a secured server via an internet connection	Percentages; Confidence Intervals; Odds Ratios; p-values	2 years (data collection)	Moderate
34	Wright et al. (2020)	Auckland, New Zealand	Community Midwives; Women (antenatal and postnatal)	Midwives (N = 5); Women (N = 20)	Hospital	To assess the acceptability and feasibility of the Maternity Case-finding Help Assessment Tool (MatCHAT), a tool designed to provide e-screening and clinical decision support for depression, anxiety, cigarette smoking, use of alcohol or illicit substances, and family violence among pre- and post-partum women under the care of midwives Helping to understand the acceptability and feasibility of digital screening; helping to understand the barriers and enablers to digital screening	MatCHAT app; included brief smoking, drinking and other drug use questions; the Patient Health Questionaire-2 (PHQ-2) for depression, with the full Patient Health Questionaire-9 (PHQ-9) triggered when PHQ-2 positive; an anxiety question triggering the General Anxiety Disorder-7 (GAD-7) when positive; and four questions regarding family violence	Mixed Methods Research; Co-design; Quantitative and Qualitative components; Grounded Theory; general inductive approach	Semi-structured interviews; data collection via MatCHAT app program via a web link included numbers of screens completed, positive cases, participants who wanted help and the level of care recommended, and ratings of acceptability, feasibility and utility from online surveys	Descriptive statistics; general inductive approach to thematic analysis of Qualitative themes	8-months	Low

**Table 2:** Summary table of effectiveness, feasibility and acceptability of digital screening in pregnancy and postpartum

ID	Author/Year	Measure	Method/Data Analysis	Comparison Group
3	Diez-Canseco et al. (2018)	Effectiveness	Quantitative: descriptive analyses, frequencies and percentages; Qualitative: Interviews	No
6	Drake et al. (2014)	Effectiveness	Cronbach’s α; Thematic Analysis	No
9	Flynn et al. (2011)	Effectiveness	Cronbach’s α; Quantitative Analysis: Pearson correlations; Comparative AUC for ROC contrasts between EPDS and PHQ	No
10	Fontein-Kuipers & Jomeen (2019)	Effectiveness	Quantitative Analysis; proportion of maternal distress; reliability analysis of Whooley questions; diagnostic accuracy of Whooley items for depression, trait-anxiety, pregnancy-related anxiety; population prevalence of maternal distress; ROC analysis of EDS, STAI and PRAQ-R2 at T1 & T1 (Q1 &2)	No
14	Guevara et al. (2016)	Feasibility Acceptability	Summary statistics on the number of eligible parents, depression screens administered, and positive screens by site were collected; Differences in proportions by site using chi- square statistics; Assessed for trends in the monthly proportion screened using a chi- square test of trend statistic; Thematic Analysis	No
15	Guintivano et al. (2018)	Effectiveness	Descriptive statistics; State-level birth rate data; ICC’s to measure test–retest reliability for continuous variables; Binomial tests to measure agreement for binary variables; Squared weighted Cohen’s kappas to measure test–retest reliability for categorical variables	No
16	Hahn et al. (2021)	Feasibility Acceptability	Univariate analysis (χ^2^, N and p-value) of the first cohort; Logistic regression coefficients; Socio-demographic variables; birth complications; subjective birth-related trauma; PMS; postpartum blues; stressful life events; breastfeeding; within- and out-of-sample validation study design	Yes—three distinct groups: women with PPD, women with Adjustment Disorder (AD), and healthy controls (HC)
18	Highet et al. (2019)	Effectiveness	Cronbach’s α (EPDS administered digitally); Participant characteristics; psychosocial risk (n & %); mean screening time; rates of depression and anxiety	No
19	Jiménez-Serrano et al. (2015)	Effectiveness	Machine Learning (ML); Pattern Recognition (PR); Naive Bayes Model; Logistic Regression; artificial neural network (ANN); support vector machines (SVM)	Yes – PPD and no PPD
21	Kallem et al. (2019)	Effectiveness	Bivariate analyses (Chi-square and t test) were conducted comparing the maternal and infant factors of mothers who completed the EPDS and did not complete the EPDS; Multivariate logistic regression was used to estimate maternal and infant clinical and sociodemographic factors that predict service use	Yes – women who received services and women who did not receive services
22	Kim et al. (2007)	Feasibility Acceptability	Quantitative outcomes of interest were completion rates for the IVR screening and the percentage of women with mild to severe depressive symptoms. Research outcomes included reports of patient satisfaction (n & %) with the system along with their preferences for an intervention	No
23	Kingston et al. (2017)	Effectiveness Feasibility Acceptability	Adapted version of Renker and Tonkin’s tool of feasibility and acceptability; ITT analysis; Baseline differences in groups were compared using independent t tests (means) and chi-square tests (%); Descriptive data (frequencies and 95% CIs; means and SDs) to describe the sample	Yes – women who completed paper-based screening compared to E-screening
25	Marcano-Belisario et al. (2017)	Feasibility	Completion times (median, mins, secs); proportion; median; chi-square; sample sizes and percentages	No
27	Poleshuck et al. (2015)	Feasibility Acceptability	Analytic plan—growth curve analysis; quadratic effects; cross-sectional mean differences using ANCOVA; moderation effects; latent class analysis	No
28	Quispel et al. (2012)	Effectiveness	Cronbach’s α coefficient; intraclass correlation coefficient, Cohen’s κ and Kendall’s τ-b. Criterion validity NPV; PPV secondary measure; risk profiles and to describe feasibility judgements they used conventional descriptive and comparative statistics; Posthoc Bonferroni adjusted pair wise comparisons were performed to identify any group related difference; Power 0.80 and p value < 0.05	No
29	Martinez-Borba et al. (2019)	Feasibility Acceptability	Descriptive analysis of the sample; Analysis of dropout rates (proportion of women who completed each assessment in relation to women who were registered into the program); Exploration of women’s usability reports and satisfaction with HM	No
30	Shore et al. (2020)	Effectiveness	Descriptive analyses on patient characteristics, process measures and outcome measures (%, N, χ^2^,df, p-value)	No
31	Tsai et al. (2014)	Effectiveness Feasibility Acceptability	Cronbach’s α coefficient; Pearson correlation coefficient; calculating sensitivity, specificity, and likelihood ratios using standard formulas; ROC curves, calculating the area under the ROC curve (AUC) using the trapezoidal rule and comparing AUC values using the algorithm	No
32	Willey et al. (2020)	Feasibility Acceptability	Thematic analysis – inductive and deductive approach; saturation of themes; hybrid approach to thematic analysis was utilised	No
34	Wright et al. (2020)	Feasibility Acceptability	Descriptive statistics; general inductive approach to thematic analysis of Qualitative themes	No

###  Data extraction using TDF

#### Step 1: Data extraction

- First review author (JC) independently identified and extracted information from the included qualitative and mixed-methods studies about women’s and HCPs perceptions and experiences of digital screening for mental health. Extracted data from 12 of those studies were recorded in an Excel spreadsheet (one spreadsheet per study). Each data point was categorised as either (1) raw data (e.g., participant’s quotations from qualitative studies); (2) analysed data from the results sections (e.g., thematic analysis) or (3) interpretive descriptions and summaries from results.
  Information from the included studies with a qualitative component were in the form of single quotes, several quotes or paragraphs deemed appropriate within a particular author’s key theme regarding digital screening. Data extracted from the studies included key themes and sub-themes in regards to digital screening. Specifically, it included the author’s interpretation or description of the key theme (verbatim author interpretation), the quote (with page number of article) and if the extracted data was considered a barrier, enabler or both to digital screening.

#### Step 2: Data coding

- Extracted data was deductively coded (i.e., mapped) to the TDF by author JC according to the TDF domain and construct that they were determined to represent. A TDF Coding Manual developed by authors JC and JB assisted with identifying the key domains, constructs, barriers and enablers to digital screening for mental health in pregnancy and postpartum at a theoretical level.
- Step 3: Data checking
  A second author (MS) independently reviewed all of the included qualitative and mixed-methods studies to determine author agreement and consensus on the TDF coding using the TDF Coding Manual. Any disagreement or uncertainty was discussed until 100% consensus was reached.
- Step 4: Presentation of findings
  Results from the TDF domain coding of the included full-text articles are presented in Table 3. The TDF domains and constructs were counted in frequencies to reflect their importance within those categories and key themes and may include single or multiple TDF domains and constructs (Atkins et al. 2017).

#### Step 5: Recommendations

- Recommendations for the implementation of digital screening for mental health in pregnancy and postpartum were developed using Michie et al.’s (2008) matrix which maps theoretical domains (e.g., behavioural determinants) to effective behaviour change using an expert consensus, combined with the TDF domains (Cane et al. 2012). The authors used their multi-disciplinary clinical and research experience to provide recommendations most relevant to the settings found to address the barriers and enablers identified by the research presented in the systematic review (Table 4).

**Table 3:** TDF mapping of key themes regarding digital screening for mental health in pregnancy and postpartum

TDF Domain (number of barriers and enablers) and Key Theme	TDF Constructs (number of barriers and enablers)	Barriers/Enablers	Sources	Statements & Quotes
Knowledge (N = 37) Knowledge of digital screening	Knowledge (N = 28) Procedural Knowledge (N = 5) Knowledge of task environment (N = 3)	Enabler (E)	Doherty et al. (2018); Drake et al. (2014); Dyurich and Oliver (2020); Gance-Cleveland et al. (2019); Guevara et al. (2016); Johnsen et al. (2018); Pineros-Leano et al. (2015); Willey et al. (2020); Wright et al. (2020)	Knowledge of digital screening by women and HCP’s and the value of screening on a digital platform, including feedback from both women and Healthcare Professionals “I like having it as an Epic (EHR) alert and making it easy to click... because then it’s nice to have the family do it without you in the room so that you can address it after the fact. I think people are a lot more honest...” (p. 1865) (Guevara et al. 2016 – Knowledge – Procedural Knowledge) (E) “I would definitely recommend..., because I had a really good experience from the screening program... it's really good. I would definitely say the screening program is very good...” [Farzana] (p.e432-433) (Willey et al. 2020 – Knowledge – Knowledge) (E) “I thought it was pretty easy” (p. 309) (Drake et al. 2014 – Knowledge – Procedural knowledge) (E)
Skills (N = 22) Skills of the Healthcare Professional and women	Skills (N = 1) Skills development (N = 3) Competence (N = 0) Ability (N = 4) Interpersonal Skills (N = 10) Practice (N = 1) Skills assessment (N = 1)	Enabler (E)	Barry et al. (2017); Doherty et al. (2020); Doherty et al. (2018); Guevara et al. (2016); Johnsen et al. (2018); Pineros-Leano et al. (2015); Willey et al. (2020); Wright et al. (2020)	Skills of the Healthcare Professionals and women to competently complete the digital screening or participate in professional development and education to further their knowledge “It probably builds the relationship between the parent and the provider more than it does anything else. They know you care about them too.” (p. 1865) (Guevara et al. 2016 – Skills – Interpersonal Skills) (E)
Social/professional role and identity (N = 40) Social professional role and identity of Healthcare Professional	Professional identity (N = 0) Professional role (N = 29) Social identity (N = 0) Identity (N = 0) Professional boundaries (N = 2) Professional confidence (N = 10) Group identity (N = 0) Leadership (N = 0) Organisational commitment (N = 0)	Enabler (E) Barrier (B)	Barry et al. (2017); Diez-Canseco et al. (2018); Doherty et al. (2020); Doherty et al. (2018); Gance-Cleveland et al. (2019); Guevara et al. (2016); Johnsen et al. (2018); Pineros-Leano et al. (2015); Wright et al. (2020)	Social professional role and identity of Healthcare Professionals ability to do their job effectively, requirements of their job and belief that digital screening is part of their role Case manager, nurse: ‘… have all of our documentation done in one place, rather than double-documenting.’ (Focus group 2) (p.213) (Pineros-Leano et al. 2015 – Social/professional role and identity – Professional role) (E) Participants also stressed, however, that they did not want technology to replace “that personalised touch,” becoming an “avenue for the midwife to cut short the interaction with a patient,” which “defeats the purpose” (M3) (p.6) (Doherty et al. 2020 – Social/professional role and identity – Professional role) (B) “... she told me a story about herself, about her own pregnancy... this wasn’t at all what I needed. I needed the two of us to talk about me and to discuss what I had written about my concerns in the questionnaire.” (Mary, Int. 5) (p. e110) (Johnsen et al. 2018 – Social/professional role and identity – Professional boundaries (B) Other women shared the impression that “a midwife is not a mental health professional” (PW8). For PW3, sharing data related to her mental health would prove valuable only if her midwife “has received training, and when I’m talking about training, I’m talking about therapeutic training, about how to handle with care the data.” Both women and midwives highlighted the power-dynamics implicit in data sharing; “she knew so many things about me, I didn’t want to share everything [emphasis] with her” (M2). PW3 was keen to avoid a mode of interaction driven by scores and thresholds, “You scored 10 out of 10, good one!’ I don’t want to have this kind of chat with my midwife.” (p.5–6) (Doherty et al. 2020 – Social/professional role and identity – Professional confidence) (B)
Beliefs about capabilities (N = 4) Beliefs about capabilities of Healthcare Professional and women	Self-confidence (N = 4) Perceived competence (N = 2) Self-efficacy (N = 0) Perceived behavioural control (N = 1) Beliefs (N = 1) Self-esteem (N = 0) Empowerment (N = 6) Professional confidence (N = 0)	Enabler (E)/Barrier (B)	Barry et al. (2017); Doherty et al. (2020); Doherty et al. (2018); Dyurich and Oliver (2020); Pineros-Leano et al. (2015)	Beliefs about the capabilities of women and Healthcare Professionals to complete digital screening “It’s about empowering women to take responsibility for their mood and contacting us”; “It’s a risk assessment on whether that woman or client needs additional support” (p.2714) (Barry et al. 2017 – Beliefs about capabilities – Empowerment) (E/B)
Beliefs about consequences (N = 8) Beliefs about consequences for Healthcare Professional and women	Beliefs (N = 1) Outcome expectancies (N = 2) Characteristics of outcome expectancies (N = 0) Anticipated regret (N = 4) Consequents (N = 3)	Barrier (B)	Barry et al. (2017); Doherty et al. (2020); Johnsen et al. (2018); Wright et al. (2020)	Beliefs about the consequences for women and Healthcare Professionals with completing digital screening “I think you’d find it quite hard to be honest about that [the EPDS], if you knew your midwife was seeing it” (PW2), “because the language is quite clinical…I will think twice before replying to it” (PW3), “I don’t want them to think that I’ve got depression, because then that means it would go on my record, it might affect whether they believe I can look after my baby…it would affect my level of honesty I think, in reporting” (PW7). (p.5) (Doherty et al. 2020 – Beliefs about consequences – Outcome expectancies; Anticipated regret) (B) Midwives to become “more focused on my self-reports as opposed to maybe signs that she should notice…if she notices me sobbing for something silly, then that’s her cue that ‘maybe I should ask her about her mental health’” (M2). (p.6) (Doherty et al. 2020 – Beliefs about consequences – Consequents) (B)
Goals (N = 1) Goals for women during pregnancy and postpartum	Goals (distal/proximal) (N = 1) Goal priority (N = 0) Goal/target setting (N = 0) Goals (autonomous/controlled) (N = 0) Action planning (N = 0) Implementation intention (N = 0)	Barrier (B)	Johnsen et al. (2018)	Goals during pregnancy and postpartum for women “... what could I write? I need a purpose... perhaps if you are a soft romantic you wish for a good pregnancy... And who am I writing to? So no.” (Lisa, Int. 5) (p. e108) (Johnsen et al. 2018 – Goals – Goals (distal/proximal) (B)
Memory, attention and decision processes (N = 3) Memory, attention and decision processes for women and Healthcare Professionals	Memory (N = 2) Attention (N = 0) Attention control (N = 0) Decision making (N = 1) Cognitive overload/tiredness (N = 0)	Enabler (E)	Doherty et al. (2020); Guevara et al. (2016); Pineros-Leano et al. (2015)	Use of digital screening to enhance memory and attention and assist in decision making for depression and anxiety assessment cut-off scores, referral and treatment services M3 envisioned the use of a mobile application as a means of overcoming cognitive limitations; “I might have forgotten what happened two weeks ago…if they would retrieve it and then say ‘Oh you mentioned this…this thing happened, do you want to share more?" (p.5) (Doherty et al. 2020 – Memory, attention and decision processes – Memory (E) *A mother in the study suggested that the app could be seen as a means of overcoming her cognitive limitations when recalling her emotions over the previous weeks*
Environmental context and resources (N = 69) Environmental context and resources required for digital screening	Environmental stressors (N = 1) Resources/material resources (N = 46) Organisational culture/climate (N = 4) Salient events/critical incidents (N = 0) Person and environment interaction (N = 17) Barriers and facilitators (N = 0)	Barrier (B) Enabler (E)	Barry et al. (2017); Diez-Canseco et al. (2018); Doherty et al. (2020); Doherty et al. (2018); Drake et al. (2014); Dyurich and Oliver (2020); Gance-Cleveland et al. (2019); (2016); Guevara et al. (2016); Johnsen et al. (2018); Pineros-Leano et al. (2015); Willey et al. (2020); Wright et al. (2020)	Environmental context and resources required to complete digital screening, including available and accessible technology (e.g., computer, tablet, mobile phone), privacy, room, staff, finances, organisational support, pressure, time and any difficulties or special requirements needed for women in order to complete screening Some days I was screening and I was really excited to do it, but when I was with the third pregnant woman screened, I looked at my watch and it was already 10:30 am, and by noon I had to see 12 women. That meant that I had to stop using the tablet and rush to finish on time with all 12 consultations from my shift. [Midwife, antenatal care service] (p.9) (Diez-Canseco et al. 2018 – Environmental context and resources – Person and environment interaction) (B) “On the app it is quicker to get it done... you actually have a physical result” (Ms. Blue). (p. 47) (Dyurich and Oliver 2020 – Environmental context and resources – Person and environment interaction) (E) Ms. Green “liked the fact that it would tell me how I was doing fast,” and was respectful of their privacy, “without it being intrusive or anything like that, because you know with the app it is very comfortable.” (p. 47) (Dyurich and Oliver 2020 – Environmental context and resources – Person and environment interaction & Resources/material resources (E) ‘…we need a system that’s going to make it simple and quicker and effective and that follows on if you want services to actually act on what you found…’ [Midwife F] (p. 268) (Wright et al. 2020 – Environmental context and resources – Resources/material resources) (E) ‘…The more improved screening is, the more numbers we can say, well look this is the number of women that we’ve got, now you need to give us more resources. We can actually use this as a tool for getting those resources.’ [Midwife F] (p. 269) (Wright et al. 2020 – Environmental context and resources – Resources/material resources) (E)
Social influences (N = 27) Social support for women	Social pressure (N = 0) Social norms (N = 0) Group conformity (N = 0) Social comparisons (N = 1) Group norms (N = 0) Social support (N = 28) Power (N = 0) Intergroup conflict (N = 0) Alienation (N = 0) Group identity (N = 0) Modelling (N = 0)	Enabler (E)	Diez-Canseco et al. (2018); Doherty et al. (2020); Drake et al. (2014); Dyurich and Oliver (2020); Dyurich and Oliver (2020); Gance-Cleveland et al. (2019); Guevara et al. (2016); Johnsen et al. (2018); Willey et al. (2020); Wright et al. (2020)	Social support for women during pregnancy and postpartum, including referral through digital screening and treatment for depression and anxiety “... in particular I remember one mother who was really pleased that we had asked about her mental state... and I know she participated in the [MH referral] program. And she really found it beneficial.” (Guevara et al. 2016 – Social influences – Social support) (E) Women also clearly related the value of data sharing to the severity of their own need; “if a person is asking for help…wants some help…it’s really useful. Whereas if a person is doing fine it’s like ‘oh, why are you intruding on my space’” (M3) (p.5) (Doherty et al. 2020 – Social influences – Social support (E)
Emotion (N = 51) Use of digital screening to express emotion	Fear (N = 4) Anxiety (N = 14) Affect (N = 29) Stress (N = 1) Depression (N = 10) Positive/negative affect (N = 0) Burn-out (N = 0)	Enabler (E)/Barrier (B)	Barry et al. (2017); Diez-Canseco et al. (2018); Doherty et al. (2020); Doherty et al. (2018); Drake et al. (2014); Dyurich and Oliver (2020); Gance-Cleveland et al. (2019); Guevara et al. (2016); Johnsen et al. (2018); Willey et al. (2020)	Use of digital screening as a tool for women to express their emotions during pregnancy and postpartum “if you don't ask it, you don't tell,... you don't open it up...You... keep it inside and, build it up, like a solid something inside your body. And when you open it up maybe you might need help with something,..., when you have the chance to express people know what your needs are and then they might be able to help you and guide you and advice you. And I think it's really good.” [Aung] (p.e423) (Willey et al. 2020 – Emotion – Affect) (E) As Ms. White noted, “I’ve been more overwhelmed, so I took it... it gave me a sad face.” (p. 47) (Dyurich and Oliver 2020 – Emotion – Depression) (E) “If it’s a tool to elicit their true feelings, then that’s only going to be good isn’t it?” (p.2714) (Barry et al. 2017 – Emotion – Affect (E/B) Participants described how accurately “the app has been reflecting my feelings” (Ms. Mustard). (p. 47) (Dyurich and Oliver 2020 – Emotion – Affect (E)
Behavioural regulation (N = 12) Self-monitoring of behaviour using validated psychological assessments and action planning	Self-monitoring (N = 10) Breaking habit (N = 0) Action planning (N = 1)	Enabler (E)	Doherty et al. (2018); Drake et al. (2014); Dyurich and Oliver (2020); Gance-Cleveland et al. (2019); Johnsen et al., (2018); Willey et al. (2020)	Use of digital screening as a tool for women and Healthcare Professionals to self-monitor women’s behaviour using validated psychological assessments and action planning Ms. White described her reaction to the first time the EPDS yielded a higher score: “So I had to kind of take a step back and think what am I doing? What’s going on?” She described the adverse effects of lack of insight and stated, “[depression] happens without you knowing it” and indicated the app was helping her achieve self-awareness because “you are keeping an eye on yourself.” (p.46) (Dyurich and Oliver, 2020 – Behavioural regulation – Self-monitoring) (E)

The TDF domains and constructs of Optimism, Reinforcement and Intentions have been omitted from Table 3 as no information from the Systematic Review was mapped to them; Enabler (E); Barrier (B)

**Table 4:** Best practice recommendations for implementation of digital screening for mental health in pregnancy and postpartum

Behavioural determinant (TDF Domain) (Cane et al. 2012)	Behavioural change techniques (Michie et al., (2008)	Examples to support Healthcare Professionals (HCP’s)	Examples to support women
Knowledge	Information regarding behaviour, outcome	Organisations to use standarised and valid mental health assessment tools for digital screening in pregnancy and postpartum and follow appropriate recommended national/clinical guidelines	Provide information to women about digital screening for mental health in pregnancy and postpartum, including description of questions and terminology
Skills	Goal/target specified: behaviour or outcome; Increasing skills: problem solving, decision making, goal setting; Rehearsal of relevant skills	Organisations to provide adequate training and education sessions on digital screening for mental health for HCP’s, regarding appropriate use, scoring and interpretation	
Social/professional role and identity	Social processes of encouragement, pressure, support	Organisations to encourage appropriate communication with HCP’s to understand the importance of including digital screening for mental health as part of their role Encourage HCP’s to provide appropriate professional and interpersonal support to women during digital screening, using women-centred communication skills	Provide information to women so that they understand the role the maternity HCP has in screening for mental health and management
Beliefs about capabilities	Increasing skills: problem solving, decision making, goal setting; Social processes of encouragement, pressure, support; Self-monitoring	Organisations to provide adequate training, education sessions and support on digital screening for mental health so that HCP’s can believe in their capabilities to perform it as part of their role	Encourage and empower women to participate in digital screening and to take responsibility for monitoring their own mental health during pregnancy and postpartum
Beliefs about consequences	Persuasive communication; Information regarding behaviour, outcome; Self-monitoring; Feedback	Provide training for HCP’s to communicate effectively with women about the purpose of digital screening and the benefits for women	Provide information to women about the benefits of digital screening for mental health
Goals	Increasing skills: problem solving, decision making, goal setting	HCP’s to encourage women to set realistic and achievable goals for digital screening and mental health treatment	Encourage women to set realistic and achievable goals for digital screening and mental health management
Memory, attention and decision processes	Increasing skills: problem solving, decision making, goal setting; Planning, implementation; Prompts, triggers, cues	HCP’s to be aware of appropriate cut off scores for digital screening mental health assessments and diagnoses of mental health issues	Provide information to women that digital screening can help keep track of their mental health before, during pregnancy and postpartum Provide information to women about cut off scores for digital screening assessments and mental health diagnoses
Environmental context and resources	Environmental changes (e.g., objects to facilitate behaviour), time management	Organisations to provide appropriate technology (e.g., FHIR; use of tablets and mobile phones) for digital screening for mental health to allow for easy flow of women’s results, information and referral through EMR/EHR for HCP’s Organisations to provide appropriate staffing to facilitate the digital screening (e.g., HCP – Midwife, Nurse, Clinician; GP; Doctor) Organisations to provide manageable workload for HCP’s Provide women with appropriate advice and support to complete digital screening effectively (e.g., use of Interpreter or Patient Navigator) Provide women with technological support if issues arise during digital screening Provide women with options on how the information is presented to them (e.g., layout of digital screening – scrolling or paging screen layout; method of digital screening (e.g., Web or mobile phone application) Organisations to advise HCP’s as to the appropriate time(s) to screen women (prenatal/1st/2nd/3rd trimester during pregnancy/postpartum) Allow women sufficient time to complete the digital screening Allow women safe and private facility to complete digital screening (e.g., home, waiting room, appointment room) Ensure mental health assessment for digital screening is available in various language translations and formats (both written and audio format) Send link to digital screening to women prior to maternity clinic visit Applications to send push notifications to women to reduce technical difficulties Record information in a check box on EMR/EHR regarding referral to mental health services Inclusion of electronic alerts in EMR/EHR as point of care reminders to prompt HCP’s to complete digital screening with women Organisations to provide continuity of staff and location to women if applicable	Provide women with access to appropriate technology (e.g., use of tablets, mobile phone or computers) to complete digital screening Provide information to women regarding support available to explain digital screening or provide technical assistance (e.g., information sheet at commencement of screening; assistance of HCP, Interpreter or Patient Navigator) Encourage women to complete digital screening in a safe and private environment Encourage women to allow sufficient time to complete digital screening
Social influences	Social processes of encouragement, pressure, support	Encourage appropriate referral, support and treatment pathways for women following completion of digital screening for mental health	Provide women with appropriate referral, support and treatment pathways specific to their mental health needs during pregnancy and postpartum
Emotion	Stress management; Coping skills	Organisations to provide HCP’s with training, education and support to encourage women to provide accurate responses regarding their emotions during pregnancy and postpartum	Encourage women to be accurate in their responses to regarding their emotions during pregnancy and postpartum
Behavioural regulation	Planning, implementation; Prompts, triggers, cues, monitoring, self-monitoring	HCP’s to provide information to women about the importance of screening at regular time points during their pregnancy for behavioural self-monitoring for mental health and allow for effective action planning	Provide information to women and encourage them to understand the need for regular behavioural self-monitoring for mental health during pregnancy and postpartum and allow for effective action planning

Key: HCP = Healthcare Professionals; FHIR = Fast Healthcare Interoperability Resources (application programming interface for exchanging electronic health records); EMR = Electronic Medical Record; EHR = Electronic Health Record; Note: The TDF domains and constructs of Optimism, Reinforcement and Intentions have been omitted from Table 4 as no information from the Systematic Review was mapped to them

## Results

**Fig. 1:**
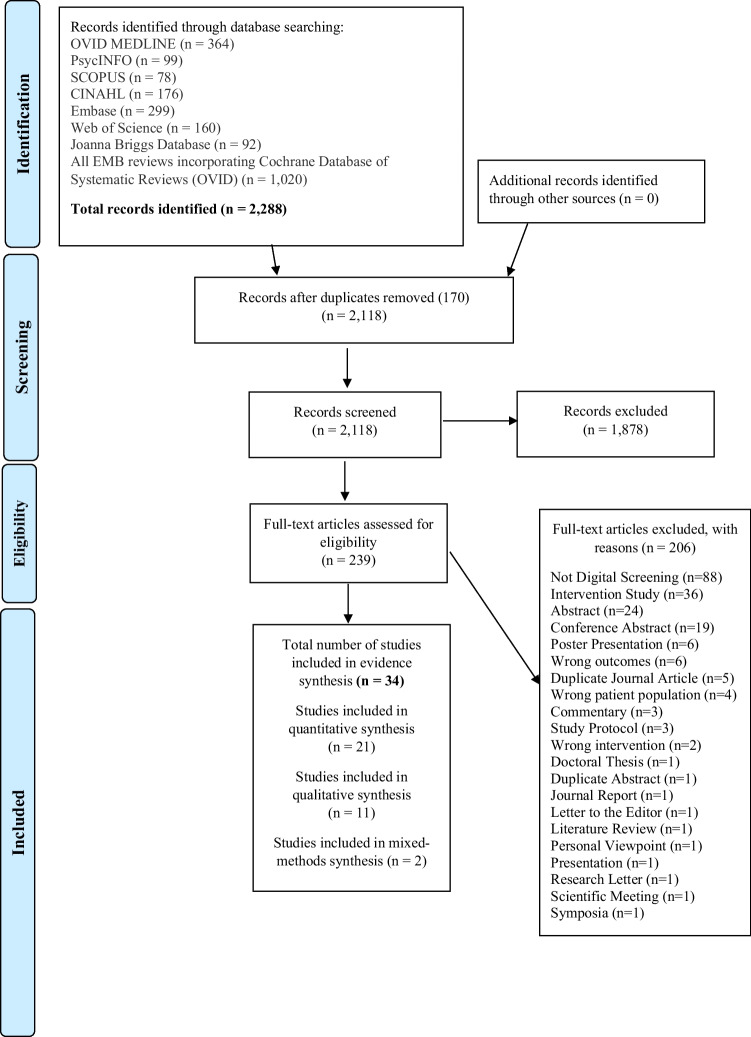
PRISMA flow diagram of systematic review

Database searching retrieved 2,288 relevant articles. These studies were imported into Endnote reference management software (Thomson Reuters 2020) and filtered for duplication. Studies were then transferred into Covidence systematic review software for screening (Veritas Health Information, 2020), where further duplicate articles were removed, leaving 2,118 articles to be screened. There were 1,878 studies excluded after title and abstract screening and 206 studies removed after full-text screening, resulting in a final number of 34 papers (PRISMA Flowchart, Fig. 1).

### Results

Table 1 displays a simplified summary of the study characteristics of the 34 included full-text articles of the systematic review recorded from the data extraction Excel spreadsheet. Table 2 displays a summary table of effectiveness, feasibility and acceptability. Table 3 displays the mapping of the articles to the TDF (Cane et al. 2012). Table 4 displays the best practice recommendations for the implementation of digital screening for mental health in pregnancy and postpartum, with examples to support both HCPs and women across different healthcare settings.

### Risk of bias assessment

Of the 34 included studies, nine were determined to be low risk, eight were low/moderate risk and seventeen articles with moderate risk of bias (Table 1). A total of eleven studies employed a qualitative study design, twenty-one studies employed a quantitative design and two studies used a mixed-methods design (e.g., qualitative and quantitative). Of the quantitative studies, thirteen used a cohort study design (e.g., longitudinal and the participants were selected on the presence or absence of a risk factor), three were cross-sectional design, three were randomised controlled trials, one was a non-randomised controlled trial and one used a pre-test post-test study design.

Of the qualitative studies, four were determined to be low risk and seven were low/moderate risk of bias. The studies were adequately descriptive in nature, with clear outcomes and justifications for the research methodology. Several had no or limited information (e.g., Gordon et al. 2016; Doherty et al. 2018) on whether their assumptions had been adequately explored (e.g., consideration of own role, potential bias and influence during the study), however, this was not considered to be a major flaw given the information the articles reported.

Of the 21 quantitative studies, five were determined to be low risk, one was low/moderate risk of bias and 15 were moderate risk of bias. Limited information was provided in some studies both in regards to the blinding of outcome assessors to the exposure and to what percentage of individuals were not included in their results. Most of the quantitative studies followed cohorts of women over an extended period of time.

Of the two studies that were of mixed-methods study design, both were determined to be of moderate risk of bias. Limitations included inconsistencies in the reporting of the quantitative and qualitative components of the studies (Guevara et al. 2016; Diez-Canseco et al. 2018).

Key

EDS: Edinburgh Depression Scale.
EDS-10: Edinburgh Depression Scale (10 questions).
EPDS: Edinburgh Postnatal Depression Scale (EPDS-2; EPDS-3; EPDS-5; EPDS-7).
EPDS-10: Edinburgh Postnatal Depression Scale (10 questions).
EPDS (online): Online version of EPDS (e.g., completed online using the Internet and a Laptop).
EPDS-lifetime version: Edinburgh Postnatal Depression Scale (21 questions).
BrightSelf: BrightSelf mHealth Application.
EMA: Ecological Momentary Assessment (5 questions—Mood, Sleep, Worry, Enjoyment and Energy).
SRQ (WHO): Self-Reporting Questionnaire – World Health Organisation.
mHealth: mobile health.
Ginger.io Application: mobile phone application (downloaded to smartphone).
PHQ-2: Patient Health Questionnaire (2 questions).
PHQ-9: Patient Health Questionnaire (9 questions).
GAD-2: General Anxiety Disorder (2 questions).
GAD-7: General Anxiety Disorder (7 questions).
EMR: Electronic Medical Record.
EHR: Electronic Health Record.
Global Health Scale: Assess the participant´s mental and physical health as well as pain, fatigue, social connections, and overall health and quality of life (10 questions).
AUC: Area Under the ROC Curve.
ROC Curve: Receiver Operating Characteristic Curve.

Whooley Questions (2 questions).

Arroll Question (1 Question).

STAI: State-Trait Anxiety Inventory (20-items).
STAI-S: State-Trait Anxiety Inventory (state subscale – 20 items).
STAI-T: State-Trait Anxiety Inventory (trait subscale – 20 items).
PRAQ-R/PRAQ-R2: Pregnancy-related Anxiety Questionnaire revised (10-items).
PPAQ: Pregnancy Physical Activity Questionnaire (32 activities).
SBIRT: Screening, Brief Intervention, Referral to Treatment (SBIRT) framework.
AAS-2: Abuse Assessment Screen (2 items).
NIDA Quick Screen: National Institute on Drug Abuse (Quick Screen).
AUDIT-C: Can help identify patients with alcohol misuse (3-question screen).
Pre-Pregnancy BMI: Pre-pregnancy Body Mass Index.
GWG: Gestational Weight Gain.
GTT: Glucose Tolerance Test.
Godin Shepard: Godin Shephard Leisure-Time Physical Activity Questionnaire.
Insomnia Severity Index: Insomnia Severity Index outcome measure.
Tablet-based: Using a i-Pad; iPad computer.
iOS App: Mobile Operating System (Apple Inc.)
PMD: Perinatal Mental Disorders.
PPD: Post-partum Depression.
iCOPE: Digital Screening Platform (Centre for Perinatal Excellence).
EPQ-N: Eysenck Personality Questionnaire – Neuroticism (12-items).
EPQ-R: Eysenck Personality Questionnaire – Revised (48-items).

eDPPMobile Application developed by Jiménez-Serrano et al. (2015).

ML: Machine Learning.
PR: Pattern Recognition.
ANN: artificial neural network.
SVM: support vector machines.
ICC: Intraclass correlation coefficients.
WHO-5 Wellbeing Index: World Health Organisation – Wellbeing Index (5-questions).
Cambridge Worry Scale: 16-item questionnaire assessing worry.
IVR: Interactive Voice Response (technology).
ALPHA: Antenatal Psychosocial Health Assessment (15 risk factors).
MINI: Mini International Neuropsychiatric Interview.
ITT: Intention to Treat Analysis.

**Renker & Tonkin’s Assessment of the feasibility of computerised screening for interpersonal violence (9-items).**
Questback: Anonymous Online Questionnaire administered by Questback (http://www.questback.com).
IPTW: inverse probability of treatment weighting.
CI: Confidence Interval.
NHS: National Health Service.
NICE: National Institute for Health and Care Excellence.
Snap Mobile App: Snap Mobile Application (for Apple iOSTM running on Apple iPad Air and Apple iPad mini tablet computers. Responses were stored in Snap WebHost.
RCT: Randomised Controlled Trial.
ANCOVA: Analysis of Covariance.
Promote W: Electronic Psychosocial Screening and Referral Tool (standardised screening tools).
WHO-QOL: World Health Organisation’s WHOQOL-BREF Scale (26-item version of the WHOQOL-100 assessment).
Patient Navigator: Person who assists patient in a clinic environment to navigate the health care system and needs of patients.
GyPsy: GyPsy (in Dutch), derived from Gynecology and Psychiatry).
PDA: Personal Digital Assistant (self-report screening; hand-held computer).
HM-Web & HM-App: HappyMom Web & App versions.
ERQ: Emotion Regulation Questionnaire (10-items).
CAE: Cuestionario de Afrontamiento del Estrés = Stress Coping Questionnaire (42-items) (Martinez-Borba et al. 2019).
QLI: Quality of Life Index (33-items).
SRSS: Social Readjustment Rating Scale (43-items).
ODK: Open Data Kit Software (android application).
MUAC: Mid upper arm circumference.
HemoCue Hb 301 system: Haemoglobin concentration.
Household Food Insecurity Access Scale: Household food insecurity.
IPV (HITS assessment): Intimate Partner Violence (Hurt, Insult, Threaten and Scream).
MSSS: Maternity Social Support Scale.
GHS: Global Health Scale.
WAST: Women’s Abuse Screening Test (5-items).
HFIAS: Household Food Insecurity Access Scale (9 questions).
MPAS: Maternal Postnatal Attachment Scale (19 items).

### Overall summary

Twelve studies examined the effectiveness of digital screening (Studies 3,6,9,10,15, 18,19,21,23,28,30,31). Eight studies explored the acceptability and feasibility of digital screening for mental health in pregnancy and postpartum (Studies 14,16,22,25,27,29,32,34). Some studies explored effectiveness, feasibility and acceptability (e.g., Study 23; Kingston et al. 2017). The remaining 14 studies explored the lived experiences of women and healthcare professionals, the prevalence of depression and anxiety among women, mental health care use and referral for services, the development of digital screening tools and the implementation and effectiveness of digital screening within healthcare systems (Table 1).

### Method and type of digital screening

Overall, the total sample size across the 34 included studies included 32,859 participants. Screening methods varied with completion primarily via a tablet, computer or mobile phone application. The EPDS (Cox et al. 1987) was the main psychological assessment used to assess depression and anxiety symptoms, with 20 of the 34 included studies using some form of the EPDS as their primary psychological assessment (e.g., EPDS-10; EPDS-lifetime version; validated for specific cultures). The PHQ-2 (Löwe et al. 2005) or PHQ-9 (Kroenke et al. 2001) was also commonly used (*n* = 11), often in conjunction with the EPDS or other assessment measure as they were found to be more feasible and easier to implement.

Many researchers developed their own applications, with adaptive features, with some applications focused on the personal subjective experience of women and encouraged support-seeking behaviours, such as prioritising the midwife-client relationship (BrightSelf-App; Diez-Canseco et al. (2018). Others focused on the ability to screen the patients for depression and other concerns (MatCHAT; Wright et al. 2020), and undertake risk prediction (eDPP Predictor; Marcano-Belisario et al. (2017). Some had the ability to screen women in multiple languages and produce both clinical and patient reports (iCOPE; Highet et al. 2019) and one enabled responses to vocal prompts using Interactive Voice Response (IVR) technology (Kim et al. 2007). Other applications were able to screen and manage symptoms of perinatal depression and promote wellness during pregnancy (VeedaMom; Dyurich & Oliver (2020) and some provided screening and advice (GyPsy Screen and Advice; Quispel et al. (2012) & iCOPE (Highet et al. 2019).

One study assessed the relationship between the survey layout (e.g., paging or scrolling on the app) and screening with scrolling resulting in a slightly faster completion time (median = 4 min 46 s) than a paging layout (median = 5 min 33 s) (Marcano-Belisario et al. 2017). Another study compared platforms web (HappyMom-Web) or mobile (HappyMom App; HM-App; downloaded app) longitudinally over pregnancy and post-partum. A higher proportion of women responded at each time point to the HappyMom Web sample (27.3–51.1%), compared to the HappyMom-App sample (9.1–53.1%), possibly because involvement was supported by HCPs for the web-based program. However, whilst longitudinal retention was low for both it was slightly higher for the app (9.1%) compared to the web platform (4.6%) (Martinez-Borba et al. 2019).

### Effectiveness of digital screening for mental health in pregnancy and postpartum

The 12 studies (3,6,9,10,15,18,19,21,23,28,30,31) that assessed the effectiveness of digital screening reported effectiveness in detecting and referring women for mental health treatment in pregnancy and postpartum, with good internal consistency (Cronbach’s α = 0.88–0.90, Quispel et al. 2012; Cronbach’s α = 0.81, Drake et al. 2014; Cronbach’s α = 0.89, Highet et al. 2019). It was also effective when compared to paper-based screening, measured with an adapted version of Renker and Tonkin’s tool of feasibility and acceptability (Kingston et al. 2017) and in different languages (Quispel et al. 2012; Tsai et al. 2014; Diez-Canseco et al. 2018; Fontein-Kuipers & Jomeen 2019; Kallem et al. 2019).

### Acceptability and feasibility of digital screening for mental health in pregnancy and postpartum

There were eight studies that assessed the acceptability and feasibility of digital screening for mental health. Digital screening for mental health was acceptable and feasible (Martinez-Borba at el., 2019) to both women (Hahn et al. 2021; Kim et al. 2007; Marcano-Belisario et al. 2017; Poleshuck et al. 2015; Willey et al. 2020 and Wright et al. 2020) and HCPs (Guevara et al. 2016; Wright et al. 2020). Digital screening was found to be acceptable across cultures and countries (e.g., North America, United Kingdom, Spain, Australia), healthcare settings (e.g., Public Health, Community Health Clinics, Antenatal Clinics, Hospital Settings) and using various delivery options, suggesting generalisability of the results to the wider population (Table 1).

Research in a community maternal and child health setting found that completing the EPDS and Psychosocial Questions on a tablet enabled women to complete screening themselves in a timely manner, with reduced scorer error (e.g., reverse scoring of EPDS items; Matthey et al. 2012) and 100% accuracy. An automated tailored plain language report sent to women by SMS or email reported risks and directed relevant health information and available health services. This facilitated the health professional consultation and supported self-management at home. Clinical summaries prompted referrals when required and provided scores saving time for health professionals enabling more time for discussion with women (Highet et al. 2019). Screening of parents post-partum over 20-months in the USA by Guevara et al. (2016) utilised both paper-and-pencil and electronic versions of the PHQ-2 within EHR that incorporated electronic screening alerts and a check box for service referrals. The use of electronic alerts reminded clinicians when to screen patients, facilitated screening and included suggested language for explaining the results to parents. Use of alerts increased screening from 12.8% of eligible parents to 54.5% and interviews with clinicians identified that alerts were of benefit in reminding them when screening was due and that the electronic discussion points and automatic scoring of the depression tool facilitated screening (Guevara et al. 2016).

Kallem et al. (2019) found digital screening completed as part of routine care at the 2-month well child check beneficial and effective in identifying women at risk of mental health concerns. Mothers completed the screening via a tablet in the waiting room, with the results of the screen presented in the child’s EHR.

Table 2 displays a summary table of the effectiveness, feasibility and acceptability of digital screening in pregnancy and postpartum. Table 3 displays the TDF mapping of key themes regarding digital screening for mental health in pregnancy and postpartum.

### Barriers and enablers to implementation for digital screening in pregnancy and postpartum

Results of the systematic review were mapped to the TDF (Cane et al. 2012) to identify barriers and enablers, as well as key themes (Table 3). The three main TDF domains identified included Social/professional role and identity, Emotion and Environmental context and resources.

#### Social/professional role and identity

Social/professional role and identity refers to HCPs ability to do their job effectively, including the requirements of their job and the belief that digital screening is a part of their role, which enables them to implement it effectively. The most prominent constructs included professional role, professional confidence and professional boundaries. Key barriers were the ability to which the HCP’s thought that digital screening was part of their role and what it consisted of on a daily basis (Pineros-Leano et al. 2015), the level of confidence that the HCPs or the women had in their ability to complete digital screening effectively (Doherty et al. 2020) and if the women felt that their HCP’s were acting appropriately within their professional boundaries (Johnsen et al. 2018).

#### Emotion

Emotion refers to the use of digital screening as a tool for women to help express their emotions during pregnancy and postpartum, which was considered a key enabler. The most prominent constructs were affect, anxiety, depression, fear and stress. This was reflected in the ability for a digital screening platform to encourage women to identify, label and express their emotions effectively during the pregnancy and postpartum period (Barry et al. 2017; Dyurich and Oliver 2020; Willey et al. 2020) and seek further knowledge (e.g., watching videos) and social support.

#### Environmental context and resources

Environmental context and resources were key barriers to digital screening. They highlighted the importance of the environment in which a woman completed the digital screening and the resources provided by the healthcare professionals and organisations. The most prominent constructs were resources/material resources and person and environment interaction. This was reflected in the importance of the availability and accessibility of technology (e.g., computer, tablet, mobile phone) (Pineros-Leano et al. 2015; Gance-Cleveland et al. 2019), room (i.e., available or separate), available staff, finances (i.e., organisations or women), organisational support and workload pressure to complete many digital screening assessments (Diez-Canseco et al. 2018).

### Discussion

This systematic review explored the acceptability, feasibility and effectiveness of digital screening for mental health in pregnancy and postpartum, as well as barriers and enablers to the implementation and best practice recommendations for future clinical practice.

### Acceptability, feasibility and effectiveness

The review found good evidence that digital screening for mental health in pregnancy and postpartum is acceptable and effective for women and HCPs and is feasible to undertake in clinical practice, providing a better alternative to standard care (e.g., paper-based screening; Kingston et al. 2017), across a variety of cultures and healthcare settings. Valid and reliable screening measures, with the EPDS (Cox et al. 1987) being the primary assessment measure of choice were able to be completed using digital platforms with accuracy by both women or HCP’s. Digital screening provided quicker administration time, increased screening capacity, reduced scoring error, generated clinical and patient reports and prompted referrals for the treatment of depression and anxiety (Highet et al 2019). The choice of user interface (app or web-based) may influence the implementation and uptake of the digital screening. However, these studies were for women completing screening at home at multiple time points and the relevance for screening in a clinical context may not apply (Martinez-Borba et al. 2019).

### Barriers to implementation of digital screening in pregnancy and postpartum and best practice recommendations for future clinical practice

Barriers to the implementation of digital screening included skills and social/professional role and identity of HCPs. This related to their role in screening and identifying women with anxiety or depressions but also their role in using digital platforms. It is important to support HCPs to increase their knowledge of digital screening, through education, training, clarity around the scope of their practice and time constraints (Bayrampour et al. 2018) as well as supporting HCPs less literate in technology and for non-regular staff unfamiliar with the technology. However, research has found digital screening and assessment is favourable and comfortable among midwives and women in general (Schmied et al. 2020) and particularly during the COVID-19 pandemic (Martin-Key et al. 2021). HCPs should reassure women regarding their beliefs about the consequences of completing digital screening, such as outcome expectancies and anticipated regret through information provision.

Environmental context and resources can also provide barriers to the implementation of digital screening, with the main area of concern being the resources/material resources and the person and environment interaction. Key barriers at an organisational level include the lack of available technology and increased workload for HCPs. Women who completed the digital screening did not find many barriers to technological issues, however, issues that were encountered were overcome with assistance from staff at healthcare facilities. Women from Culturally and Linguistically Diverse (CALD) backgrounds (e.g., people who come from different countries across the world) experienced some difficulty in responding to questions on the digital screening platforms, with feelings of being uncomfortable, uncertainty of questions and embarrassment with question content (Willey et al. 2020).

Environmental context and resources are a pivotal component in the implementation of digital screening. Organisations play an important role in the effective implementation through the resources provided to complete digital screening, such as access to the digital technology used and availability of technology (e.g., iPads for women), technological support, choice of assessment measure, availability of assessment in different languages and formats (e.g., written & audio), choice of how the assessment measure is displayed, funding, how the referrals are recorded within the EHR/EMR healthcare systems and the use of electronic alerts to prompt clinicians to complete digital screening. An important consideration is the staffing within organisations and the workload required of HCPs to conduct digital screening with women if it is not self-completed. Consideration should be made by organisations as to which application they choose, any adaptations needed, requirements for local service users and any initial and ongoing costs.

### Enablers to implementation of digital screening in pregnancy and postpartum and best practice recommendations for future clinical practice

Enablers to the implementation of digital screening include the knowledge it provides, timely self-completion, no scorer error, referral for social support, identification of emotions and the ability for women to self-monitor their own behaviours and emotions. Overall, women were able to complete digital screening effectively, with limited technological issues. They also found it particularly beneficial when the screening was available in their own language as it was more convenient, they were able to understand the questions more easily and were more truthful in their responses. Digital screening resultedin less embarrassment and improved privacy and supported equity among women and across cultures (Willey et al. 2020) or when completed by themselves through the use of Interactive Voice Response (IVR) technology (e.g., Kim et al. 2007), allowing women to self-enter their responses in a private clinic room.

For some women with decreased literacy, it was suggested that an audio format would further assist equity in access to screening (Willey et al. 2020). Women were receptive to being asked about their mental health state and the social support provided through referral either through digital screening or resources provided by HCPs. Digital screening allowed women to express their emotions, disclose mental health concerns, develop self-awareness and insight through self-monitoring (Dyurich & Oliver 2020). Recommendations to support women include providing them with information about digital screening, encouraging the development of realistic and achievable goals, providing appropriate support and referral pathways, adequate time for completion of digital screening and the provision of technical support if required. As best-practice guidance changes, it is possible that digital screening may be a more agile mode and adapt faster than paper-based screening. Clinical judgement is also used where indicated to assess for other conditions and the effectiveness of screening for other mental health conditions is beyond the scope of this paper.

### Strengths and limitations

Limitations of the review involved the exclusion of particular study designs that may have been beneficial to include in the review, such as entirely algorithm-based digital screening. However, these were deemed not to be within the scope of this review, due to clinical decision support systems and machine learning. These were only included in the review if the psychological assessment was in digital format. Further, there were few studies that included women from CALD backgrounds (n = 15), limiting generalisability, as well as limited comparison groups due to small sample sizes and methodological approach chosen. Most of the digital based platforms used the EPDS. This is not surprising as it is currently the most widely used perinatal mental health screening measure, frequently recommended in clinical guidelines and translated and validated in a multitude of languages (Blackmore et al. 2022). However, there are some concerns with the use of the EPDS and its broad applicability such as for use with Indigenous women (Chan et al. 2021) and future studies of digital screening may need to explore other measures as the evidence base changes. Additionally, while a meta-analysis was originally planned, it was not feasible due to the small number of eligible studies. Finally, seventeen of the 34 included studies (50%) were at moderate risk of bias; while this is not a limitation of this review’s design, it does reflect a limitation of the existing evidence base and more high-quality studies are recommended. Strengths of the review included exploring research over a 21-year period in relation to digital screening for mental health in pregnancy and postpartum and theory-informed recommendations for both HCP’s and women.

### Future directions

This review has identified key barriers and enablers to the implementation of digital screening and also provided recommendations for clinical practice. Future research and clinical practice should add to the literature by adapting current practice and implementing digital screening for pregnancy and postpartum in their specific healthcare settings worldwide (e.g., public, private or community), utilising the theory-informed best practice recommendations presented in this systematic review and the use of various language translations and formats. Development of new technologies (e.g., Fast Healthcare Interoperability Resources—FHIR) and mobile phone applications, including choice of layout and user interface, will be beneficial to the digital screening field for mental health in pregnancy and postpartum.

## Conclusion

Digital screening provides an innovative, acceptable, feasible and effective method to screen women for mental health concerns such as depression and anxiety in the pregnancy and postpartum period. It is effective and acceptable to women and HCPs and feasible to implement in clinical care. Important enablers include support for women to understand the role and benefits of screening and provide technological assistance, as well as providing HCPs education and training about screening, how to use the digital technology and management for women at risk. Digital screening provides the opportunity for behavioural regulation through self-monitoring and empowering women to take an active role in their mental health care, referral and treatment. The provision of appropriate organisational resources and staffing is critical, enabling widespread usage, equity and access to mental health support for women around the world during the perinatal and postpartum period.

## Data Availability

Available upon request to senior author via email.

## Code availability

N/A.

## Declarations

### Search strategy for ovid medline

Ovid MEDLINE(R) and Epub Ahead of Print, In-Process, In-Data-Review & Other Non-Indexed Citations, Daily and Versions(R).

### Special Thanks

To Dr. David Butler of the Cairnmillar Institute for his research support and guidance - JC.

### Dedication

For RH, for always inspiring me and my love of Psychology - JC.
